# IVC Measurements in Critically Ill Patients with Acute Renal Failure

**DOI:** 10.1155/2017/3598392

**Published:** 2017-09-05

**Authors:** Rami Jambeih, Jean I. Keddissi, Houssein A. Youness

**Affiliations:** University of Oklahoma Health Sciences Center, Oklahoma City, OK, USA

## Abstract

**Objective:**

To determine whether the inferior vena cava (IVC) measurement by bedside ultrasound (US-IVC) predicts improvement in renal function in patients with acute kidney injury (AKI).

**Design:**

Prospective observational study.

**Setting:**

Medical intensive care unit.

**Patients:**

33 patients with AKI were included.

**Intervention:**

US-IVC was done on admission. The patients' management was done by the primary teams, who were unaware of the US-IVC findings. Two groups of patients were identified. Group 1 included patients who were managed in concordance with their US-IVC (potential volume responders who had a positive fluid balance at 48 h after admission and potential volume nonresponders who had an even or negative fluid balance at 48 hours after admission). Group 2 included patients in whom the fluid management was discordant with their US-IVC.

**Measurements and Main Results:**

At 48 hours, Group 1 patients had a greater improvement in creatinine [85% versus 31%, *p* = 0.0002], creatinine clearance (78 ± 93% versus 8 ± 64%, *p* = 0.002), and urine output (0.86 ± 0.54 versus 0.45 ± 0.36 ml/Kg/h, *p* = 0.03).

**Conclusion:**

In critically ill patients with AKI, concurrence of fluid therapy with IVC predicted fluid management, as assessed by bedside ultrasound, was associated with improved renal function at 48 hours. This trial is registered with* ClinicalTrials.gov registration number*: NCT02064244.

## 1. Introduction

Bedside ultrasonography in the critical care setting has expanded dramatically over the last decade. It is used routinely for procedural guidance and is currently utilized for the rapid identification and evaluation of multiple conditions, including pleural disease, respiratory failure, and shock [1–3]. However, studies have not confirmed that its use has been associated with better clinical outcome.

Numerous studies demonstrating the utility of ultrasonography in the hemodynamic assessment of ICU patients have been published. In spontaneously breathing patients, the inferior vena cava (IVC) diameter and the IVC Collapsibility Index (IVC-CI) have been shown to correlate with the volume status and central venous pressure (CVP) [4, 5]. Intravascular volume depletion is likely when the IVC-CI is >50% [6]. In mechanically ventilated patients, the IVC Variation Index (ΔIVC) correlates with volume responsiveness. Volume responsiveness is likely when the ΔIVC is ≥12% in these patients [7].

Patients with acute prerenal failure and volume overload manifest a particularly challenging diagnosis. This subset of patients has signs of extravascular volume overload such as lower extremities swelling and pulmonary edema; yet they may have intravascular volume depletion or overload leading to decreased effective renal perfusion, low fractional excretion of sodium (FeNa), and increased blood urea nitrogen (BUN), creatinine, and BUN/creatinine. While a low FeNa and high BUN/creatinine are not specific for prerenal azotemia and are affected by concurrent use of medications such as diuretic treatment, a more reliable method of assessing intravascular volume status in these patients is needed.

To our knowledge, the IVC size assessment by bedside ultrasonography has not been previously used to evaluate or manage patients with acute kidney injury (AKI) in the intensive care unit.

## 2. Hypothesis

In patients with AKI, fluid management in correlation with IVC predicted management, as assessed by bedside ultrasound, will be associated with improved renal function.

## 3. Methods and Materials

### 3.1. Study Design

This was a prospective observational study conducted at the Oklahoma City VA healthcare system and the University of Oklahoma Health Sciences Center (OUHSC) between February 2014 and June 2015 (ClinicalTrials.gov number NCT02064244). The protocol was approved by the OUHSC institutional review board (IRB #3464) and a written informed consent was obtained from all patients.

### 3.2. Settings and Participants

All patients who presented to the intensive care units with a diagnosis of AKI (defined as a 1.5-fold increase in the plasma creatinine level compared to baseline) [8] were included. Baseline creatinine was defined as the last available creatinine value prior to admission. Since fluid administration may affect the IVC measurement, patients who received more than 2 L of fluids between the creatinine and IVC measurements were excluded. Other exclusion criteria included age < 18 years, chronic hemodialysis or ongoing continuous renal replacement therapy (CRRT), obstructive uropathy, pulmonary emboli, and absence of informed consent.

### 3.3. Methods

A two-dimensional echocardiographic sector using the SonoSite visceral probe P-21 (5–1 MHZ) (FUJIFILM SonoSite, Bothell, WA) was used to visualize the long axis of the IVC at the subcostal window. The M-mode was used to generate a time-motion record of the IVC diameter approximately 2 cm caudal to its junction with the right atrium. The maximum (IVC max) and minimum (IVC min) diameters of the IVC over a single respiratory cycle were collected.

In spontaneously breathing nonventilated patients, the IVC-CI was calculated as (IVC max − IVC min)/IVC max. In mechanically ventilated patients, we calculated the ΔIVC as (IVC max − IVC min)/[0.5 × (IVC max + IVC min)] while the patient was breathing comfortably, without any significant patient-ventilator asynchrony and using a tidal volume of 8–10 ml/kg of ideal body weight [6, 7]. We used the IVC-CI (≥50%) and ΔIVC (≥12%) to classify patients as potential candidates for fluid administration in spontaneously breathing and mechanically ventilated patients, respectively.

The management of the 33 patients including the use of diuretics, fluids, or dialysis was done by the primary intensive care team without knowledge of the results of the ultrasound measurements.

Two groups of patients were identified. Group 1 included patients who were managed in concordance with their IVC measurements:

- Spontaneously breathing patients with an IVC-CI ≥ 50% and mechanically ventilated patients with an ΔIVC ≥ 12% who had a positive fluid balance at 48 hours after admission
- Spontaneously breathing patients with an IVC-CI < 50% and mechanically ventilated patients with an ΔIVC < 12% who had an even or negative fluid balance at 48 hours after admission.

Group 2 included the patients in whom the fluid management was discordant with the IVC measurement.

Demographic data and baseline characteristics were recorded. These included past medical history, primary admission diagnosis, hemodynamic parameter, laboratory parameter, and echocardiographic parameters. Baseline, day 0 (inclusion day), and day 2 data were collected for all participants.

Fluid balances as well as the plasma creatinine level at 48 hours after admission were recorded.

The primary outcome was defined as the percentage of patients with improved creatinine level at 48 hours in groups 1 and 2. Secondary outcomes included changes in creatinine, creatinine clearance, and urine output.

If patients were started on hemodialysis or CRRT, they were considered as if their creatinine did not improve. For the secondary outcomes, in patients who had to start hemodialysis or CRRT, the highest known creatinine measurement was used as the 48 h value.

### 3.4. Statistical Analysis

Assuming that 2/3 of the patients will be in group 1, and assuming an improvement in the serum creatinine in 75% of group 1 and 25% of group 2 patients at 48 hours, 22 patients in group 1 and 11 patients in group 2 were needed to reject the null hypothesis with a power of 0.8, and type I error probability of 0.05.

Data is presented as mean ± standard deviation and percentages. Chi-square and Student's *t*-test were used for comparison of categorical and continuous variables, respectively. Data was analyzed for normality using the method of Kolmogorov and Smirnov. The Mann–Whitney test was used for nonparametric data.

## 4. Results

A total of 35 patients consented to participate in the study. Two patients were excluded from the final analysis due to missing data in one patient and death immediately following recruitment in another patient.

Twenty of the remaining 33 patients (60%) were classified as group 1, while 13 (40%) patients were classified in group 2 (Table 1). The baseline characteristics of these patients are summarized in Table 2.

There was no significant difference in the baseline characteristics of both groups including age, sex, body mass index, medical history, admission and final diagnosis, creatinine level, creatinine clearance, and mechanical ventilation requirement. The mean arterial pressure was lower in group 2 (76 ± 6 versus 83 ± 13,* p* = 0.04) although the number of patients on pressors was not statistically different.

In spontaneously breathing patients, the mean IVC-CI index was 57.4 ± 4.6% in group 1 compared to 31.1 ± 16% in group 2 (*p* < 0.0001). In mechanically ventilated patients the mean ΔIVC was 55.5 ± 49% in group 1 compared to 5.2 ± 4% in group 2 (*p* = 0.11) (Table 3).

At 48 hours, group 2 had a higher cumulative fluid balance than group 1 (5.8 ± 4.6 versus 4 ± 6.3 L;* p* = 0.027) (Table 4).

In the first 48 hours, CRRT was started in 2 patients in group 1 and one patient in group 2.

### 4.1. Primary Outcome

Serum creatinine improved in 17/20 (85%) patients in group 1 compared to 4/13 (31%) patients in group 2 (*p* = 0.0002) (Table 4).

### 4.2. Secondary Outcomes

Compared to day 0, there was a significant improvement in the renal function in group 1 compared to group 2 in terms of the mean change in creatinine (−0.8 ± 0.78 versus 0.39 ± 0.81 mg/dl,* p* < 0.0001), percent change in creatinine (−33 ± 26 versus 12 ± 39%,* p* = 0.0003), creatinine clearance (31 ± 39 versus 6 ± 34 mL/min,* p* = 0.0048), and percent changes in creatinine clearance (78 ± 93 versus 8 ± 64%,* p* = 0.002) (Table 4).

On day 2, the urine output was better in in group 1 compared to group 2 (0.86 ± 0.54 versus 0.45 ± 0.36 ml/Kg/hour;* p* = 0.03).

## 5. Discussion

AKI is a common diagnosis in the ICU, involving 13% to 78% of admissions [9, 10]. Accurate assessment of the fluid status is essential to its management. Its pathophysiology involves multiple mechanisms, including hypovolemia and various types of shock. Although fluid loading can be helpful by restoring the renal perfusion pressure in many hypovolemic patients with prerenal failure, it may be deleterious in hypervolemic patients with renal hypoperfusion related to a low cardiac output, where further fluid loading can lead to pulmonary edema and deterioration in oxygenation [11]. Moreover, fluid overload in ICU patients have been shown to be an independent risk factor for development of AKI, as well as the 28-day mortality in these patients [12].

In the present study, we demonstrated that one-third of AKI patient managed in the ICU may receive inappropriate fluid therapy. Although the IVC measurements were not used to guide fluid therapy, this study is the first to report an improvement in renal function when the fluid management correlated with that predicted by the ultrasonographic assessment of the IVC. To our knowledge the primary outcome of all prior studies was the change in cardiac output (measured by echocardiography or by thermodilution) following a fluid challenge.

In patients with spontaneous ventilation, respiratory variations are highly variable. An IVC-CI ≥ 50% has been shown to strongly correlate with hypovolemia and low CVP in critically ill spontaneously breathing patients [4, 13]. In 2012, Muller et al. reported that a cut off of 40% had the best ROC curve for predicting volume responsiveness measured by an increase in echocardiographic cardiac output of at least 15% [14].

We elected to choose a cut off of IVC-CI ≥ 50% for managing fluid therapy in spontaneously breathing patients, as it may indicate the presence of significant reserve in tolerating fluid therapy. All our group 1 patients who were spontaneously breathing (*n* = 13) had a IVC-CI of more than 50%, and 6 out of 9 spontaneously breathing patients in group 2 had a IVC-CI of less than 40%.

The main limitation of our study is related to its observational nature, and the lack of randomization. Group 2 patients had lower blood pressure. This may indicate that they were sicker with more acute tubular necrosis which could explain the lack of creatinine improvement following intravenous fluid hydration. However, both groups were similar in regard to the rest of the baseline characteristics including the number of patients on pressors, pressors dose, admission creatinine, and final diagnosis.

Only 3 patients achieved a negative fluid balance at 48 hours. Therefore, our results may not be applicable in the settings where diuresis is needed. The results of our study need to be interpreted carefully in such patients.

In summary, correlation of fluid management with guided fluid therapy based on the use of bedside ultrasound to assess IVC variation and collapsibility indices may increase the rate of improvement in creatinine and creatinine clearance in patients with AKI. More importantly, ultrasound assessment of the IVC collapsibility can identify patients with AKI who will not respond to fluid therapy. This is especially important in the medical intensive care unit where the general trend is to administer fluid. The utility of ultrasound guided fluid management in future randomized prospective trials is needed to confirm these results.

## Figures and Tables

**Table 1:** Distribution of patients according to their IVC assessment. For each category, group 1 included the patients who were managed in concordance with the ultrasound findings (italic values), while group 2 (bold values) include patients who were not managed in concordance with the ultrasound findings.

	IVC	
48-hour fluid balance	IVC-CI ≥ 50% (spontaneous breathing) ΔIVC ≥ 12% (mechanically ventilated)	IVC-CI < 50% (spontaneous breathing) ΔIVC < 12% (mechanically ventilated)
Positive (*n*)	*18*	**12**
Creatinine improved (%)	*16/18 (88)*	**4/12 (33)**

Negative (*n*)	**1**	*2*
Creatinine improved (%)	**0/1 (0)**	*1/2 (50)*

Total	19	14

**Table 2:** Baseline characteristics of the 33 patients.

	Group 1 (*n* = 20)	Group 2 (*n* = 13)	*p*
Age (y)	57 ± 12	59 ± 17	0.78
BMI (Kg/m^2^)	31 ± 14	28 ± 5	0.98
Male, *n* (%)	11 (55)	8 (62)	0.9
PMH			
Hypertension, *n* (%)	11 (55)	6 (46)	0.73
Coronary artery disease, *n* (%)	2 (10)	3 (23)	0.36
Diabetes mellitus, *n* (%)	5 (25)	6 (46)	0.27
Congestive heart failure, *n* (%)	2 (10)	5 (38)	0.08
Pulmonary hypertension, *n* (%)	1 (5)	0	1
CKD, *n* (%)	3 (15)	0	0.26
Final diagnosis, *n* (%)			
Sepsis, *n* (%)	11 (55)	10 (77)	0.28
Pneumonia, *n* (%)	3 (15)	3 (23)	0.6
Congestive heart failure, *n* (%)	3 (15)	1 (8)	NS
MI, *n* (%)	0	1 (8)	0.39
DKA/HHS, *n* (%)	3 (15)	1 (8)	NS
GI bleeding, *n* (%)	2 (10)	0	0.5
Hepatic encephalopathy, *n* (%)	1 (5)	0	NS
Hemodynamic			
Mean arterial pressure (mmHg)	83 ± 13	76 ± 6	0.04
Heart rate (bpm)	99 ± 18	101 ± 20	0.78
Pressors required, *n* (%)	3 (15)	5 (38)	0.21
Prior ejection fraction (%)	61 ± 8	54 ± 19	0.42

Mechanical ventilation, *n* (%)	7 (35%)	4 (31%)	NS
TV (ml/Kg of ideal body weight)	10 ± 3	8 ± 2	0.26
Creatinine (mg/dL)			
Baseline	1.13 ± 0.85	0.8 ± 0.25	0.6
Day 0	2.88 ± 1.43	2.42 ± 1.16	0.34
Creatinine clearance (mL/min)			
Baseline	114 ± 65	122 ± 58	0.9
Day 0	42 ± 28	45 ± 25	0.78
BUN (mg/dL)	57 ± 33	49 ± 34	0.52
Na (mmol/L)	135 ± 5	135 ± 7	0.88
Albumin (g/dL)	2.93 ± 0.9	2.65 ± 0.63	0.41
BNP (pg/mL)	254 ± 227	570 ± 519	0.27
BUN/creatinine	19.9 ± 7	20.7 ± 8.9	0.77
BUN/creatinine > 20, *n* (%)	8 (40)	8 (62)	0.29
FeNa (%)^*∗*^	2.0 ± 2	1.1 ± 0.6	0.43
FeNa < 1%, *n* (%)	4/11 (36)^*∗*^	2/4 (50)^*∗*^	1

^*∗*^FeNa data was available for 11 patients in group 1 and 4 patients in group 2.

**Table 3:** Baseline ultrasound data in the 33 patients.

	Group 1 (*n* = 20)	Group 2 (*n* = 13)	*p*
IVC min	0.9 ± 0.7	1.5 ± 0.7	0.026
IVC max	1.7 ± 0.6	1.9 ± 0.6	0.31
Spontaneously breathing patients, IVC-CI (*n*)	13	9	<0.0001
Mean (%)	57.4 ± 4.6	31.1 ± 16	
Range (%)	52–67	12–54	
Mechanically ventilated patients, ΔIVC (*n*)	7	4	0.11
Mean (%)	55.5 ± 49	5.2 ± 4	
Range (%)	4–125	0–8	
Potential fluid responsive per IVC, *n* (%)	18 (90)	1 (8)	<0.01

**Table 4:** Renal function at baseline, day 0, and day 2 in the 33 patients.

	Group 1 (*n* = 20)	Group 2 (*n* = 13)	*p*
Creatinine (mg/dl)			
Baseline	1.13 ± 0.85	0.8 ± 0.25	0.6
Day 0	2.88 ± 1.43	2.42 ± 1.16	0.34
Day 2	2.0 ± 1.5	2.8 ± 1.6	0.18
Mean change in creatinine (mg/dl)	−0.80 ± 0.78	0.39 ± 0.81	<0.0001
Change in creatinine (%)	−33 ± 26	12 ± 39	0.0003
Percent of patients with improved creatinine	17 (85)	4 (31)	0.0002
Creatinine clearance (mL/min)			
Baseline	114 ± 65	122 ± 58	0.9
Day 0	42 ± 28	45 ± 25	0.78
Day 2	73 ± 52	51 ± 46	0.2
Mean change in creatinine clearance	31 ± 39	6 ± 34	0.0048
Percent change in creatinine clearance	78 ± 93	8 ± 64	0.002
Urine output at day 0, ml/kg/h	0.63 ± 0.83	0.32 ± 0.28	0.66
Urine output at day 2, ml/kg/h	0.86 ± 0.54	0.45 ± 0.36	0.03
Change in urine output (day 0 to day 2) (ml/kg/h)	0.32 ± 0.8	0.11 ± 0.5	0.47
Fluid balance (day 2) (L)	4 ± 6.3	5.8 ± 4.6	0.027
